# Pre-operative physiotherapy following unilateral ankle fractures at a tertiary hospital in South Africa: Perceptions of patients and nurses

**DOI:** 10.4102/sajp.v77i1.1501

**Published:** 2021-01-12

**Authors:** Sabeeha Dangor, Prithi Jayaraman-Pillay, Stacy Maddocks, Verusia Chetty

**Affiliations:** 1 Department of Physiotherapy, Faculty of Health Science, University of KwaZulu-Natal, Durban, South Africa

**Keywords:** ankle fractures, nurse, pre-operative physiotherapy, prehabilitation, ankle fractures, nurse, South Africa

## Abstract

**Background:**

Ankle fractures are a common injury because of an increase in levels of physical activity, as well as senescence worldwide. Ankle fractures often require surgical management for optimal stabilisation. Pre-operative physiotherapy is necessary to prepare patients for early mobilisation and home discharge. There is a lack of information on the influence of pre-operative physiotherapy on post-operative rehabilitation success, as well as timeous discharge, in patients with ankle fractures.

**Objectives:**

To explore the perceptions of patients receiving pre-operative physiotherapy care following a unilateral ankle fracture and the perceptions of nursing staff managing these patients at a tertiary hospital in South Africa.

**Method:**

A descriptive qualitative design, using semi-structured interviews, including both patients with unilateral ankle fractures and nurses caring for these patients, was adopted. Interviews were recorded and verbatim transcriptions were analysed utilising thematic analysis.

**Results:**

Four overarching themes emerged: the perceived benefits of pre-operative physiotherapy; inhibitors to physiotherapy rehabilitation; hidden enablers to pre-operative physiotherapy and future initiatives for rehabilitation.

**Conclusion:**

The perceived benefits included improved functional independence and safety of patients, as well as reduced burden of care for nurses. Patients also believed that pain and fear were two inhibitors to physiotherapy. Furthermore, nurses identified that organisational limitations, such as short-staffing and inadequately trained staff, inhibited pre-operative physiotherapy and continuity of care. Early post-operative discharge was a crucial hidden enabler to the pre-operative physiotherapy protocol. Recommendations included improved health education; the potential role of nursing staff as facilitators in pre-operative rehabilitation and regular, pre-operative in-patient monitoring of physiotherapy intervention.

**Clinical implications:**

Health education was perceived to have improved patient safety and compliance which subsequently reduced patient safety incidences as well as served as a risk mitigation measure. Furthermore, gait training and muscle strengthening exercises was perceived to have resulted in safe, independent mobility to ensure prompt discharge home. Consequently, a reduced post-operative length of in hospital stay results in major cost savings per patient as well as improved access and bed availability. Future studies may need to explore the effects of pre-operative physiotherapy on post-operative success and return to pre-injury activity.

## Introduction

Ankle fractures are a common occurrence worldwide (Lin, Hiller & De Bie 2010). The ankle joint is a complex joint that supports the human body’s weight during daily activities, predisposing this joint to injury (Wire & Slane 2019). Injury is more common in populations who are at a higher risk of falling, such as the elderly and athletes (Berry & Miller 2008). The steadily growing elderly population, as well as the recent awareness regarding health and the popularity of sport, are two major contributors to the growing annual incidence of ankle fractures (Lin et al. 2010).

The Weber classification system is commonly used to classify ankle fractures (Kennedy et al. 1998). Harper (1992) explained that this scheme is based on the level of the fracture on the fibula as well as the level of disruption of the syndesmosis, and may be recognised as one of three types: Weber A, Weber B or Weber C. Weber A fractures occur as a transverse fracture of the fibula, at or below the level of the syndesmosis, without any disruption to the tibiofibular ligaments or the syndesmosis. Weber B fractures occur as a fracture of the fibula, beginning at the level of the syndesmosis, resulting in some disruption of the tibiofibular ligament. However, the mortise remains stable following reduction of the fracture. Fractures are classified as Weber C when there is a fracture of the diaphysis of the fibula, and the syndesmosis is completely disrupted (Harper 1992).

Weber A fractures are often managed conservatively, whilst Weber C fractures are managed surgically by open reduction and internal fixation (ORIF) (Donken et al. 2013). Weber B fractures are managed either surgically or conservatively, depending on the extent of injury (Donken et al. 2013). Surgery of the ankle joint aims to reconstruct the joint surfaces, protect the soft tissue and promote early post-operative return to function (Goost et al. 2014:377–388). Studies suggest that for optimal stabilisation of ankle fractures surgery should occur within 8–24 h of injury to avoid post-operative complications (Carrage, Csongradi & Bleck 1991; Hoiness & Stomsoe 2000; Konrath et al. 1995; Singh, Trickett & Hodgson 2015; Sukeik, Qaffaf & Ferrier 2010).

Trauma-related injuries requiring emergency care still remain the leading cause of admissions to public hospitals in South Africa (Van As et al. 2007). The internal 2019 mid-year Orthopaedic Consolidated Morbidity and Mortality Statistics at our study setting illustrated that, between January and June 2019, overcapacity of theatres was one of the main reasons for cancellation of surgeries (Fang 2019). From these statistics, emergency surgery admissions (2430 cases) were also shown to have caused most of the orthopaedic surgical workload between January and June 2019, compared to the 774 elective surgery admissions and the 2789 patients on the elective theatre waiting list (Fang 2019). Furthermore, this mid-year report identified that trauma cases utilised 52% of theatre time (Fang 2019).

Emergency surgery admissions contribute to the backlog of admitted patients awaiting surgeries (Van As et al. 2007). Operating theatres are monopolised by emergency trauma patients, and patients with ankle fractures requiring ankle ORIF within an 8–24 h period are then moved to the non-emergency elective surgery list. This results in the postponement of their surgeries and increased pre-operative in-patient waiting times. This was also illustrated in the mid-year report (Fang 2019).

Lengthy waiting times for elective surgeries are not unique to South Africa but are a global problem and tend to reflect struggling healthcare systems (Viberg et al. 2013). In-hospital medical complications, in particular pneumonia, are often associated with longer length of stay in hospital (Ingeman et al. 2011), as well as lengthy periods of bed rest (Teasell & Dittmer 1993). Prolonged hospital stays influence the biomedical, as well as the psychosocial, well-being of patients (Rajcoomar 2017). Additionally, the economic implications, because of the costs incurred in keeping a patient in hospital, stress the healthcare system even further (Rajcoomar 2017). Prehabilitation for orthopaedic patients has been evidenced to significantly improve pain, muscle strength (of specific muscle groups) and also reduce the length of in-hospital patient stay (Moyer et al. 2017).

A study exploring prehabilitation for orthopaedic patients by Ditmyer, Topp and Pifer (2002) explained prehabilitation as a model that initiates the recovery process pre-operatively. This model encourages pre-operative patient support to ensure that each patient functions to the best of their capability whilst waiting for their surgery. Our study also explained that prehabilitation may prevent deconditioning because of the associated pre-operative inactivity. Ditmyer et al. (2002) also suggest that prehabilitation of patients may result in better post-operative outcomes, such as improved independence and function, reduced pain as well as a better quality of life. Physiotherapists at our study setting have implemented a prehabilitation protocol for patients awaiting surgery following an ankle fracture to mitigate against increased waiting times and prolonged pre-operative hospital stays. This protocol aims to promote early, safe mobilisation with an assistive device.

Although the physiotherapy intervention at our study setting has been implemented over the past seven years, the experience of the patients and staff members involved in the programme has not yet been explored. Thus, the purpose of our study was to explore the perceptions of patients receiving pre-operative physiotherapy treatment following unilateral ankle fractures. Furthermore, we aimed to understand the perceptions of the nursing staff managing these patients in the orthopaedic admission wards at a tertiary hospital in South Africa.

## Methodology

A qualitative and descriptive design was employed by conducting semi-structured interviews. This method was used in order to explore the perceptions of patients and nursing staff regarding the pre-operative physiotherapy offered to patients with unilateral ankle fractures at a tertiary hospital in Johannesburg, South Africa. This approach enabled the authors to gain an understanding of the opinions and experiences of patients receiving pre-operative physiotherapy following a unilateral ankle fracture, as well as the opinions and experiences of the orthopaedic nursing staff managing these patients (Hammarberg, Kirkman & De Lacey 2016).

Our study was conducted at a large tertiary hospital with 2888 beds, providing healthcare for a population of 3.5 million people in and around the southern Johannesburg region (Landman, Mouton & Nevhutalu 2001), which was the study context for our paper. In 2010, an audit conducted at this institution revealed that ORIFs were the most common surgical procedure performed for an ankle fracture (Pillay & Ramokgopa 2013). However, this healthcare facility is burdened by high rates of admissions without sufficient resources to meet the growing healthcare demands (Fang 2019).

Upon admission, following a unilateral ankle fracture, patients requiring surgical stabilisation have their ankle fracture supported in back slabs or plaster casts. These patients are routinely managed with a prehabilitation physiotherapy programme. The prehabilitation protocol includes pre-operative non-weight bearing mobilisation using an assistive device on level ground. Mobilisation includes stair climbing as well as transfers between different places, such as the bed and chair. Physiotherapists also provide education on the diagnosis; impending surgery; the complications of prolonged bed rest; the general safety principles for mobilisation with an assistive device and the discharge and referral process (Calatayud et al. 2016). This practice aims to assist with independence in activities of daily living in order to reduce the burden of nursing care, prevent complications associated with extended periods of bed rest and reduce in-hospital falls in preparation for early discharge, post-operatively (Lin et al. 2012).

Patients between the ages of 18 and 50 years, with a unilateral ankle fracture stabilised in a back slab, awaiting elective surgery for the fracture and admitted to the orthopaedic admission wards between 01 August 2019 and 30 September 2019, were included in our study. Nurses managing these patients, who were working in orthopaedic admission wards and had consented to participate in our study, were also included.

In-patient participants were excluded if they had sustained multiple fractures, presented with any condition that prevented pre-operative mobilisation (e.g. a plaster of Paris [POP] that was bi-valved to control swelling or if their neurovascular status was compromised), had any pre-existing co-morbidities (cerebrovascular accident, or vestibular problems) or had a history of previous admissions and/or injuries for which they were taught to ambulate by using an assistive device. Patients who were bed or wheelchair bound or were unable to comprehend and/or follow instructions because of impaired cognitive function were also excluded.

Participants meeting the inclusion criteria were selected by purposive sampling from the adult orthopaedic surgery admission wards during the period of 01 August 2019 and 30 September 2019. Participants were recruited through individual face-to-face discussions in the ward by the first author.

### Data collection

Semi-structured interviews were utilised to elicit robust and descriptive data on the perceptions or experiences of participants of pre-operative physiotherapy care. This allowed a rich narrative between the first author and the participants (Rabionet 2011). The semi-structured interview guide was designed to understand participants’ opinions on prehabilitation, their challenges with prehabilitation and suggestions for future physiotherapy. The guide was developed in consultation with experts in orthopaedics and qualitative research in tertiary academic institutes in South Africa (Rabionet 2011).

Face-to-face, in-depth, semi-structured interviews were conducted for the duration of two months for seven nurses and eight patients. Interviews lasted approximately 30–45 min. Interviews were conducted in English as participants’ language of preference. All interviews were recorded using a digital voice recorder. The first author documented the participants’ facial expressions as well as other indicators that may have added value to the data but could not be reflected in the audio files. The concept of saturation of information was used to determine the size of the sample when the in-depth semi-structured interviews with participants did not yield any new information (Merriam 1998).

### Data analysis

The interviews were transcribed verbatim following data collection. Once verbatim transcriptions of interviews were completed, member checking of the transcribed text by a third of the study participants was incorporated to ensure the accuracy and credibility of the data (Braun & Clarke 2006; Creswell 2013). The transcribed interviews were coded and thematically analysed using the Braun and Clark method of thematic analysis (Braun & Clarke 2006). The first author and co-authors familiarised themselves with the data by re-reading transcripts and journaling early impressions. Inductive open-coding was then used to organise the data collected. Codes were developed and modified by the first author and co-authors during in-depth discussions. Codes were examined and organised into themes. Themes were then reviewed and defined by the first author until consensus was achieved by all authors.

### Ethical consideration

Ethical clearance was obtained from the Human Research Ethics Committee of the University of KwaZulu-Natal, and gatekeeper approval was sought from the relevant authorities before the commencement of the study (Ethical clearance number: HSSREC/00000001/2019). Written informed consent, which included consent to audio recordings, was obtained from all participants. Participants’ anonymity was ensured by allocating research numbers.

### Trustworthiness

Two main methods were used to achieve credibility, namely triangulation and member checking. Triangulation was achieved by including semi-structured interviews with two different study groups (patients and nurses). The first author was guided by three experienced qualitative researchers throughout the entire process of data collection and data analysis to establish analyst triangulation. All interviews were recorded to ensure the accuracy of the data collected. Thorough verbatim transcriptions of randomly selected interviews were then member checked by the corresponding participants following their interview to increase the credibility of data collected (Creswell 2013). We included rich detailed descriptions of the study setting as well as participants’ perceptions. Interviews were conducted in participants’ preferred language, and thus ensured rich descriptions of their experience. Dependability ensures that our study findings remain consistent if the same study design is applied (Creswell 2013). We included comprehensive in-depth descriptions of each process involved in the data collection, the data analysis and the study findings. All documents were also kept in dated order to provide a clear audit trail so that every decision made by the first author was easily traced, which further ensured confirmability.

## Findings

Five female and two male nurses participated in our study. The mean age of the nurses was 45 years with a standard deviation of 11.5. Their ages ranged between 26 and 58 years. Of the seven nurses participating, three were enrolled nurses and four were professional nurses, of whom one was an operational manager, one a shift manager and one a community service nurse. There were five male and three female patients who met the inclusion criteria. The mean age for patients interviewed was 34.5 years with a standard deviation of 4.14, and ages ranged between 31 and 41 years. Five patients had obtained their secondary school certification, and three reported that they did not complete secondary school. All participants included were black South Africans.

Four overarching themes were identified from the interviews conducted with patients, following ORIF for a unilateral ankle fracture, and the nursing staff managing these patients (reflected in Table 1), namely *perceived benefits of pre-operative physiotherapy; inhibitors to physiotherapy rehabilitation; hidden enablers to pre-operative physiotherapy* and *future initiatives for rehabilitation*.

**TABLE 1:** Themes, sub-themes and illustrative quotes.

Sub-themes	Illustrative quotes
**Theme 1: Perceived benefits of pre-operative physiotherapy**	
Patient enablement	‘So yea, with the physio you get some exercises you can stand up and do a lot of things.’ (Patient 3, 41 years old) ‘Now (after physio) I can go to the loo (toilet) without asking anyone, I can go take a bath. I can do almost anything.’ (Patient 3, 41 years old) ‘Yes, because after the physio is seeing the patient, the patient is able to do most of the things themselves, they able to use wheelchairs to go to the toilet – we don’t need to give them urinary or bed pans.’ (Nurse 6, 26 years old) ‘I was walking to go to the toilet, bathroom, come back and go outside see my friends, may be talking with other people – climb to my beds, I was doing a lot of things.’ (Patient 6, 31 years old)
Patient self-confidence	‘I think you (the physios) are actually allaying anxiety – you are allaying anxiety to the patient.’ (Nurse 3, 45 years old) ‘So, after you teach me step-by-step how to use this thing (crutches) I was feeling safe.’ (Patient 7, 37 years old) ‘It gives me that energy that I can do my own things.’ (Patient 7, 37 years old)
Patient self-empowerment	‘When I arrive I’m stressful – thinking about my leg. Don’t feel I will walk again. Maybe the doctor will cut my leg or what. So when they come they give me hope (referring to physiotherapist). Say “no” you can walk again. When they teach me they show me something: “No you can do it.” I said, “No I can’t do it.” They say “No you can do it. Let’s try.” And I can try and I feel like, no I will be normal again. It’s just this thing is passing.’ (Patient 6, 31 years old) ‘Err. My thinking – if you did not teach me how to use this thing (referring to crutches), means I would lay on the bed. I would be using the wheelchair the whole of my life, but because of that physiotherapy it helps me, so I don’t have to use a wheelchair now.’ (Patient 7, 37 years old) ‘It actually empowered me to be on my feet again.’ (Patient 8, 35 years old)
Physical improvements	‘Even the bedsore will be limited. Because if they don’t mobilise we are causing more harm also; so the more they mobilise the more we are going far away from the bedsores and everything.’ (Nurse 2, 36 years old) ‘But after the physio and the exercising, and everything, the pain went down.’ (Patient 3, 41 years old) ‘At the start I had a lot of pain, but as I exercised the pain actually just faded away.’ (Patient 8, 35 years old) ‘It’s a good procedure because it helps the patient to strengthen the muscles. It promotes circulations. It prevents stiffness of joints by exercising them on daily basis.’’ (Nurse 1, 58 years old)
Reduced nurses’ burden of care	‘You’re really helping us, because if once the patient was mobilising, it’s easier for me not to. I am not going to exercise or, err, ask the patient to turn. I just note down, “Okay, done with physio.” Then, like, the position changed by himself and he is not lying in the same position.’ (Nurse 4, 55 years old) ‘Because if the patient is taught how to move, how to exercise, and I come and say, “Can you please move this side mama?” already, because you have already taught the patient, it will automatically be easy.’ (Nurse 3, 45 years old) ‘I think it will be more difficult on the one that hasn’t been seen by a physio (therapist), waiting to be seen by the physio (therapist). And it will be easy for the one that has been seen by the physio (therapist). Yeah, you do see that difference. So for me, it will be more work, more burden, to the one, the one that hasn’t been seen.’ (Nurse 3, 45 years old)
**Theme 2: Inhibitors to physiotherapy rehabilitation**	
Intrinsic patient limitations	‘I was scared to fall down, because (hiccup) I didn’t know how to hold it and how to use it (referring to using crutches).’ (Patient 5, 30 years old) ‘Yah. For the first time I was scared because it’s the first time I used this thing (gestures to crutches).’ (Patient 7, 37 years old) ‘Yah. For the, for the first day, yah, it was, it was difficult because I’m not sure this thing (crutches) is going to be balanced or what can happen on my legs.’ (Patient 7, 37 years old)
Extrinsic patient barriers	‘Yah, usually on the first episode on admission we provide them with bed pans because they are unable to move their legs and they are in pain, you see.’ (Nurse 1, 58 years old) ‘Ya the pain was too much (on the first day of mobilising), because, ya, I was used to my leg being like this (extends knee on injured leg, implying straight leg); so if you put it down, ya, if you hold it down (when standing), hey, it was heavy and it was paining.’ (Patient 4, 31 years old) ‘The pain was too bad at that time. And I was willing to learn how to use this, but the pain was the problem.’ (Patient 5, 30 years old)
Organisational limitations	‘… because if you (physiotherapist) are not here to, to assist them (patients), or to train them (patients), we (nurses) will be doing everything from bed one up to bed thirty-eight and it will be difficult for us as well.’ (Nurse 4, 55 years old) ‘Oh that one (ward exercises), it’s a challenge. Like our patients, we need to do more of them (ward exercises) but because of, like, shortage of staff, it’s a problem. So we end up not doing enough.’ (Nurse 5, 43 years old) ‘Well we (nurses) are not the same. Where I used to work – I used to work overseas – where I used to work before you start working in the unit, you must go for manual handling. They don’t do it here.’ (Nurse 3, 45 years old) ‘We use cot beds – as you can see you in our ward the cot bed; because of we are afraid patient may fall.’ (Nurse 6, 26 years old)
**Theme 3: Hidden enablers to pre-operative physiotherapy**	
Patients’ improved self-worth	‘Actually, helped other people next to me – they start exercising – helped them get up on their feet too. It had a good effect in my health.’ (Patient 8, 35 years old) ‘It’s very stressing when you laying down the whole time. It’s better to wake up and do something in your own and be helpful.’ (Patient 1, 32 years old) ‘For now I’m feeling good because (hiccup) I can do what I want myself. Even going outside for the sunshine and come back in here. So it’s easier for me now than the time I was just lying down.’ (Patient 5, 30 years old) ‘What you taught them – like spreading the gospel – they teach one another.’ (Nurse 1, 58 years old)
Patients’ drive for post-operative rehabilitation	‘I’m coming back right here for physio. Been given a letter, and I’ll be, I’ll be there on time.’ (Patient 8, 35 years old) ‘You’re saying that I have to go to the nearest clinic, I will go.’ (Patient 5, 30 years old) ‘Some of the patients, they leave them, but they come maybe after two days, three days, seeking them, you see (referring to physiotherapist’).’ (Nurse 1, 58 years old) ‘Because they take this, this copies to the nearest clinic when they want to go there for an exercise.’ (Nurse 1, 58 years old)
Nurse as an advocate for rehabilitation	‘I inform the patient to elevate the leg to prevent from swelling and to promote circulation.’ (Nurse 1, 58 years old) ‘We encourage the patient to at least mobilise, mobilise with physios.’ (Nurse 2, 36 years old) ‘As long as they are injured, the pain will be there irrespective of physiotherapy (isn’t it). After exercising or after a physiotherapist attended to the patient, we give them sedation or medication so they must continue.’ (Nurse 1, 58 years old)
Reduced length of stay	‘I think it’s, it’s safe to start them pre-operatively because some of them, even post-operatively, some they don’t get even chance. The doctors, they come back from theatre being discharged (example) over the weekend, and then they go home. At least they will have that bit of knowledge because they’ve practised before.’ (Nurse 4, 55 years old ‘So it will take long to mobilise them post-operatively. But if you mobilise them before operation, post-op is just a continuation. So it reduces – if you leave them prior operation, it can prolong their stay in hospital.’ (Nurse 5, 43 years old) ‘Overcrowding, it is one thing, because almost every day we are admitting patients, so if ever they stay for long it means the ward will be overcrowded. When it comes again, like, taking care of, like, manpower taking care of those patients, it will – instead of taking care of new patients, we will be taking care of patients who have been admitted for a long time. So it will put more strain to the supplies and stock.’ (Nurse 5, 43 years old)
**Theme 4: Future initiatives for rehabilitation**	
Education as part of rehabilitation	‘I personally would have liked to know about the surgery: what’s going to happen, how long it’s going to take. What are they going to put inside me. Ya, that’s what I’d like (to know from physiotherapist).’ (Patient 3, 41 years old) ‘And how I’m going to be taking long to get things come all right (referring to healing and education from physiotherapist).’ (Patient 7, 37 years old) ‘I can say, they would still be in the dark, because they don’t know anything concerning their fractures – fractured ankle and whatever. They only depended on nurses only, but physios (therapist) act as an eye opener to them.’ (Nurse 1, 58 years old)
Use of assistive devices for pre-operative rehabilitation	‘I think that it’s better to have the crutches, to learn with the crutches (referring to pre-operatively).’ (Patient 3, 41 years old) ‘I like this (gestures as crutches). That one (walking frame) is going to be difficult. If I’m go for the steps, it going to be difficult (referring to pre-ambulation pre-operatively).’ (Patient 7, 37 years old) ‘No I think the crutches are much better. Safer actually (referring to pre-operative use of crutches).’ (Patient 8, 35 years old) ‘But what I can say is that most of the patients that you have seen, you have put them on the crutches (referring to pre-operatively).’ (Nurse 3, 45 years old) ‘I think it was better to start early because I have to be used to using the crutches. So you start early, the better you get used to it.’ (Patient 5, 30 years old)
Nurse as a facilitator of pre-operative rehabilitation	‘My role in most cases, the doctors they ask me to apply maybe, hmmm, below back slab to prevent the injury from furthering.’ (Nurse 1, 58 years old) ‘I motivate the patient by teaching the patient about the diagnosis.’ (Nurse 1, 58 years old)` ‘When, when I come in the morning, I say don’t forget continue with your exercises. As long as they are waiting for you, I mean they can yah. It can make a difference.’ (Nurse 7, 54 years old)
Regular monitoring of intervention	‘Ya. You can increase (referring to monitoring) from Monday to Friday. Because this things it helps them. They enjoy this things (referring to physiotherapy).’ (Nurse 1, 58 years old) ‘To give me more training. To show me a lot of things like, sometimes you see I was washing myself in the bath. Put one leg; try to wash my body – all my body.’ (Patient 6, 31 years old) ‘Because they may need time. Maybe it’s an old man. Me, I’m still I am young. I can understand. Maybe it’s an old man. Just give him time (referring to monitoring).’ (Patient 6, 31 years old)

Theme 1, *perceived benefits of pre-operative physiotherapy,* included the sub-themes of patient enablement; patient self-confidence; patient self-empowerment; physical improvements and reduced nurses’ burden of care. Participants believed that pre-operative physiotherapy enabled patients to be more independent. It was believed to improve patient safety and afforded patients a sense of self-confidence that promoted compliance with physiotherapy management. Nurses also commented that pre-operative physiotherapy empowered patients to mobilise using an assistive device and perform activities of daily living independently, reducing the nurses’ burden of care.

The second theme, *inhibitors to physiotherapy rehabilitation,* encompassed the sub-themes of intrinsic patient limitations; extrinsic patient barriers and organisational limitations. Participants highlighted the limitations and barriers they believed would influence physiotherapy rehabilitation. Intrinsic limitations (such as fear) and extrinsic barriers (such as pain) hindered rehabilitation, according to the patients. Organisational limitations, such as limited resources or staff shortages, were also common inhibitors to the physiotherapy approach to care and the continuity of interventions.

A third theme emerged around *hidden enablers to pre-operative physiotherapy*, which included four sub-themes, namely patients’ improved self-worth; patients’ drive for post-operative rehabilitation and nurses as advocates for rehabilitation and reduced length of stay. Participants noted that patients’ improved self-worth and motivation to continue rehabilitation post-operatively were concealed enablers to pre-operative therapy. The nurses’ role in advocating for continued in-patient rehabilitation, in conjunction with pain management of patients, was also a hidden enabler to pre-operative physiotherapy. Early discharge of patients post-operatively was a further masked enabler for pre-operative physiotherapy.

The fourth theme, *the future initiatives for rehabilitation*, included the sub-themes of education as part of rehabilitation; the use of assistive devices in pre-operative rehabilitation; the nurse as a facilitator of pre-operative rehabilitation and regular monitoring of the intervention. Patients’ suggestions for future physiotherapy included receiving more information on their surgery and their recovery process. Patients also believed that introducing an assistive device during pre-operative physiotherapy assisted their functioning and rehabilitation post-operatively. Nursing staff also gave suggestions for managing pre-operative in-patient exercises, and pre-operative in-patient health education. Participants also recommended that physiotherapists should increase patient monitoring, with daily encouragement, which they believed would influence their compliance.

## Discussion

Our study sought to explore the perceptions of prehabilitated patients following a unilateral ankle fracture, as well as the experience of the nursing staff managing these patients in a tertiary hospital in South Africa. The conclusions that arose from discussions with the participants included their perceived benefits of pre-operative physiotherapy; the inhibitors to physiotherapy rehabilitation; the hidden enablers to pre-operative physiotherapy and participants’ recommendations for future rehabilitation initiatives.

The ‘benefits of pre-operative physiotherapy’ was an overarching perception, with patient enablement focused on improved self-management. Achieving independence meant that patients were able to participate in day-to-day activities, such as self-care, following physiotherapy intervention. Nursing staff agreed that following pre-operative physiotherapy, patients were able to function independently. Prehabilitation in preparation for orthopaedic surgery has been shown to encourage patients to take more responsibility for their own health, safety and activities of daily living (Ditmyer et al. 2002).

Patients also reported improved self-confidence as a result of pre-operative physiotherapy. Pre-operative physiotherapy education in our study setting emphasises safety precautions and the correct handling of assistive ambulatory devices. Nutbeam (2008) concluded that empowering individuals to make independent health decisions relies largely on understanding the potential of education. Patients in our study affirmed that information improved their self-confidence and safety and this encouraged them to mobilise independently. Furthermore, they reported that mobilising from day 1, pre-operatively, improved their confidence and encouraged early independent mobilisation.

Physiotherapists at our study site employ a tenet of the motor learning theory during gait training (Maheshwari 2018). This principle encourages repetition of a movement until the movement is automatic and there is a relatively permanent change, made by either acquisition of a new movement, or by modification of a known movement. To acquire this relatively permanent change, learning must be retained (Maheshwari 2018). Typically, retention of motor learning is assessed after 24 h to ensure sufficient time (4 to 6 h after initial practice in conjunction with a full night’s sleep) for the motor memory process (Kantak & Winstein 2012). Patients are typically gait-trained with an assistive device over 1 to 3 days, or until they have achieved the ability to ambulate safely and successfully on flat surfaces; as well as being able to climb stairs. By applying the motor learning theory, patients feel confident as crutch-walking starts to become more familiar.

The second theme concerned the inhibitors to physiotherapy rehabilitation. This included patients reportedly feeling stressed and uncertain regarding their prognosis after their injury. Some patients believed they would never be able to walk again and reported that they only tried to mobilise after being motivated by the treating physiotherapists. It is common for individuals to feel worried or fearful when facing change (Vally 2005). Hospital settings present patients with a new environment and a change in their daily routine. Furthermore, patients feel alienated because of a lack of communication with healthcare providers, which often leads to patients receiving insufficient information about their diagnosis, management plan and prognosis, ultimately magnifying their feelings of uncertainty (Haldar et al. 2017). Health education is often used to reduce patient anxiety (Spalding 2003). Nursing staff reported that pre-operative physiotherapy alleviated patients’ anxiety, which further improved patients’ confidence. These findings are supported by a study conducted to explore the influence of physiotherapists on patient autonomy and supportive behaviour (Chan et al. 2009). Patients, who stated that physiotherapy facilitated their understanding of their clinical management as well as their understanding of the reasons for their treatment, were more likely to report independent motivation and prove to have greater adherence to management (Chan et al. 2009). Furthermore, our findings suggested that patient motivation and health education were also perceived to be benefits of pre-operative physiotherapy, as they served to empower patients by educating them about their weight-bearing status, safety precautions and gait-training with an assistive device; as well as facilitating bed exercises and transfers.

Participants commented that pre-operative physiotherapy influenced various physical impairments leading to physical improvements. Some patients reported that mobilising on day 1, pre-operatively, seemed to reduce their pain. Rooks et al. (2006) studied the effect of pre-operative exercise on the functional status in patients undergoing total hip and knee arthroplasty. The study concluded that non-exercisers presented with worse function and increased pain. Furthermore, early mobilisation is known to reduce patients’ risk of complications associated with bed rest (Teasell & Dittmer 1993), particularly in conjunction with bed exercise, which prevents loss of range of movement as well as the associated muscle weakness (Dittmer & Teasell 1993). Additionally, early mobilisation of patients also encourages improved circulation, which may reduce swelling and the associated pain (Teasell & Dittmer 1993).

Nurses also believed that pre-operative physiotherapy reduced their burden of care. Nursing staff echoed that successful pre-operative physiotherapy mitigated organisational challenges such as constant short-staffing. Nurse-to-patient ratio is a challenge experienced in healthcare systems. In 2009, the Solidarity Research Institute published findings of the nurse-to-patient ratios in South Africa, which illustrated those ratios might be between 1:18 and 1:44, in comparison to the standard of 1:5. This large disproportion often compromises patient care (Aiken et al. 2002; Beurhaus, Staiger & Auerbach 2003). Studies suggest that patients who begin active early mobilisation present with improved functional status, physical function and in-patient safety (Leong, Rasnah & Chong 2017). This, in turn, puts the nursing staff at a lower risk of injury and reduces the stress levels of the nurses who would otherwise have had to transfer and lift heavy patients (Trinkoff et al. 2008). Furthermore, a study conducted by Mueller et al. (2010) reported that improved patient function reduces the workload of nurses. The nurses reported that pre-operative physiotherapy gave them more time to tend to other responsibilities.

The second theme, ‘inhibitors to physiotherapy rehabilitation’, was reflected in both patients’ intrinsic limitations and patients’ extrinsic barriers. Patients identified fear as an intrinsic limitation and pain was identified as an extrinsic barrier to physiotherapy. Patients admitted to the study setting, following a unilateral ankle fracture, initially have their ankle fracture stabilised in a back slab and are then left to lie supine, whilst elevating their affected leg until they are mobilised by physiotherapists. A study conducted by Rezaei-Adaryani, Ahmadi and Asghari-Jafarabadi (2009) states that discomfort and pain are experienced by patients lying in a supine position following medical procedures, which discourage mobility. A substantial amount of evidence corroborates the model of ‘fear-avoidance’ in patients with musculoskeletal conditions (Archer et al. 2014). This model suggests that depending on how a patient interprets their pain may lead to two pathways. When pain is thought of as non-threatening, patients are likely to continue with daily activities (Archer et al. 2014). However, pain perceived as threatening will promote anxiety and provoke pain-related fear that continues during and after the healing time (Archer et al. 2014). This leads to avoidance behaviours and lack of use, often associated with depression and physical deconditioning, which consequently propagates the pain process (Archer et al 2014; Leeuw et al. 2007). Furthermore, pain during exercise has also been described as one of the major barriers to participation in, and adherence to, physiotherapy (Jack et al. 2010).

Organisational limitations, such as resource limitations as well as staff shortages, contributed to the inhibitors of rehabilitation. Nurses often complained about the shortage of personnel impeding their encouragement of physiotherapy rehabilitation, including continuity of bed exercises as well as mobilising patients. In 2015, Hoyer et al. evaluated the barriers to early mobility of hospitalised patients in a cross-sectional study, which concluded that the overall perceived barriers identified by nurses were inadequate staffing and insufficient time to mobilise in-patients.

Resource challenges, such as high hospital cot beds, predominantly found in the male orthopaedic ward in our study setting, have also been associated with an increased risk of in-patient falls (Morse et al. 2015). Patients who attempted to mobilise before receiving physiotherapy were reported, sometimes, to have been restrained in their beds by nursing staff to maintain patient safety and prevent in-patient falls. Nursing staff explained that this was again because of the shortage of nursing staff available to facilitate safe mobility, as well as some of the nurses being inadequately trained on manual handling. Horsburgh (2004) described the use of restraints as a breach of patient autonomy; and which may only be justified in healthcare settings when applied solely in the best interest of the patient to prevent further injury or harm. Also, the lack of nurses trained to mobilise hospital in-patients has been identified as a barrier to early mobilisation in other studies (Hoyer et al. 2015).

The theme ‘hidden enablers to pre-operative physiotherapy’ emerged from patients’ improved self-worth; patients’ drive for post-operative rehabilitation; the role of nurses as advocates for rehabilitation and reduced length of stays for patients, post-operatively. Our study participants believed that pre-operative physiotherapy improved patients’ self-worth and encouraged patients’ drive and compliance with post-discharge rehabilitation. One patient also reported that he was able to help the patients next to him, and that made him ‘feel good’. Matiti (2015) conducted a study to explore patient dignity in acute care. Evidence from our study supports the positive effects of patient independence and self-worth. Patients in Matiti’s study often associated dignity with self-worth, and patients believed that a sense of independence contributed to patient dignity. Improved self-worth motivates patients to participate in pre-operative physiotherapy and adhere to the education given to them (Chan et al. 2009). Patients in our study also reported improved independence and nurses reported improved patient compliance with therapy. Patients were reported to have participated in group exercise classes at night without physiotherapists supervising them. The patients’ drive for post-operative rehabilitation was also highlighted in our findings. Nursing staff reported that most patients took their clinic referral forms with them upon discharge from in-patient care, and if they forgot their forms, they would return for the follow up-forms.

Nursing staff identified their role as advocates for rehabilitation. They reported being primarily involved in encouraging mobilisation, the education of pain management, the positioning of patients to minimise swelling and pain and administering medication and sedatives to enhance biomedical management. Pain management is a known crucial enabler to physiotherapy (Jack et al. 2010), which indirectly influences physiotherapy intervention. Length of hospital stay served as another hidden enabler to pre-operative physiotherapy. Nursing staff commented that pre-operative physiotherapy influences the patients’ length of stay. A prolonged hospital stay would affect their biomedical, as well as their psychosocial, well-being (Rajcoomar 2017). Other studies have also shown that in-patient exercise and mobility programmes in acute care hospitals may reduce hospital costs and reduce the length of stay (De Morton, Keating & Jeffs 2007; Peiris, Taylor & Shields 2011). Nurses also noted that longer hospital stays result in increased financial burdens on patients and, more so, on the healthcare facility. Another ramification of prolonged stays, expressed by the nursing staff, was ward overcrowding as a result of delayed discharges and high orthopaedic ward patient turnover. Overcrowding has often been associated with prolonged hospital stays. Furthermore, a predominant cause of overcrowding is often a lack of available beds (Boyle et al. 2012).

Patients also believed that there was insufficient time, post-operatively, for adequate rehabilitation; and nursing staff believed that delaying physiotherapy to after surgery would not be viable to ensure timely discharge. A study in 2012 conducted by Lin et al. explained that hospitals often discharge patients with insufficient instruction and minimal information because of poor communication and planning within the interdisciplinary team. Lin et al. (2012) described discharge planning as a concept that encourages an interdisciplinary approach to continuity of healthcare and a crucial link between in-hospital management and post-discharge health management. A key aspect of discharge planning is optimising patient independence in preparation for post-discharge, and ambulation is a vital part of independence (Lin et al. 2012).

The final theme concerned ‘future initiatives for rehabilitation’. Participants communicated that education should be included as part of rehabilitation. Patients also requested that future physiotherapy programmes should include more information on their surgery and the hardware used to correct or stabilise their fractures. They wanted to receive information on the post-operative healing timeline, emphasising weight-bearing status and weaning off from their assistive devices. Giraudet-Le Quintrec et al. (2003) describe how the benefits of pre-operative education from a collective multidisciplinary health education session before surgery might improve patient knowledge, reduce pain before surgery and minimise anxiety.

All participants suggested that the pre-operative physiotherapy protocol should maintain the practice of prescribing elbow crutches, as participants believed that elbow crutches are safer to use; more convenient for minibus taxi travel; easier to wean off compared to a walking frame and safer to mobilise on stairs. Elbow crutches are often prescribed for middle-aged patients with short-term injuries, because they are the easier device to wean off as they allow a two point gait pattern that closely simulates normal gait (McDonough & Razza-Doherty 1988). Physiotherapists should ideally prescribe a walking device after a complete and thorough assessment of gait, balance, cognitive ability and musculoskeletal function to encompass all the patient’s functional needs (Allet et al. 2009).

Nurses also identified their potential role as facilitators of pre-operative rehabilitation. Nursing staff suggested they could take more responsibility in ensuring patients exercise, as well as providing health education. A study conducted by Puig Ribera, McKenna and Riddoch (2005), regarding the attitudes of nurses in promoting physical activity, revealed that some barriers reported were a lack of time in their already compressed schedule and a lack of education on physical exercises. Nurses who claimed to be more interested in promoting physical activity were reported to feel qualified enough to promote physical activity, as they were found to have been self-taught on the concepts of physical activity and exercise (Puig Ribera et al. 2005).

Participants identified regular monitoring of interventions as a suggestion for future physiotherapy management. Patients recommended that physiotherapists increase patient monitoring to include more routine checks. This process was believed to potentially enable physiotherapists to better identify patients’ needs and to modify physiotherapy management accordingly: as one patient explained, he struggled to handle his assistive device correctly during some activities of daily living. Nursing staff believed that daily monitoring, as well as daily reminders to encourage patients to continue with ward exercises, might motivate patients and increase compliance. A large study conducted by Akerblad et al. (2003) supports this finding. Our study considered the effects of an educational compliance enhancement programme on treatment adherence and concluded that response to treatment increased with an educational compliance programme. Akerblad et al. (2003) described how increasing patient checks and education improves treatment adherence.

## Limitations

The perceptions of the nurses and patients were context-specific to a tertiary hospital which offers pre-operative physiotherapy for orthopaedic patients. Patients who had previously been taught to mobilise on an assistive device were excluded to enable the authors to explore the perceptions of patients gait-trained on an assistive device for the first time. Our study was limited to one study setting. A study including a health facility that does not offer pre-operative physiotherapy for orthopaedic patients, may have revealed greater insight into current practices.

## Conclusion

South African healthcare facilities are often associated with poor availability of resources, staff shortages and ward overcrowding (Bateman 2013). Long theatre waiting lists often result in lengthy pre-operative waiting times and a day-1 post-operative discharge. Establishing a protocol to optimise pre-operative physiotherapy motivated our paper. Pre-operative physiotherapy has been perceived to have many benefits to patients and healthcare providers. Benefits highlighted in our study included improved self-empowerment, self-enablement and self-confidence. It has also been shown to have perceived benefits on patients’ physical parameters, as well as reducing nurses’ burden of care.

Our findings suggest that physiotherapists should continue to adhere to the pre-operative physiotherapy protocol as it enhances patient management by alleviating patient fear and reducing pain.

Some recommendations for future physiotherapy are to improve health education, increase patient monitoring and include nursing staff as facilitators, pre-operatively. These recommendations should be further explored in practical physiotherapy approaches. Furthermore, future studies need to explore the effects of pre-operative physiotherapy for patients admitted following a unilateral ankle fracture on their post-operative success and long-term re-integration into the activities of daily living and a return to pre-injury physical activity.
